# Study on *Ajuga reptans* Extract: A Natural Antioxidant in Microencapsulated Powder Form as an Active Ingredient for Nutraceutical or Pharmaceutical Purposes

**DOI:** 10.3390/pharmaceutics12070671

**Published:** 2020-07-17

**Authors:** Tiziana Esposito, Francesca Sansone, Giulia Auriemma, Silvia Franceschelli, Michela Pecoraro, Patrizia Picerno, Rita P. Aquino, Teresa Mencherini

**Affiliations:** Department of Pharmacy, University of Salerno, Via Giovanni Paolo II 132, 84084 Fisciano (SA), Italy; tesposito@unisa.it (T.E.); gauriemma@unisa.it (G.A.); sfranceschelli@unisa.it (S.F.); mipecoraro@unisa.it (M.P.); ppicerno@unisa.it (P.P.); aquinorp@unisa.it (R.P.A.)

**Keywords:** natural antioxidants, cellular reactive oxygen species, spray-dried multi-component microsystems, enhancement of water dissolution rate, improvement of in vitro antioxidant effect

## Abstract

The administration of natural antioxidants is considered to be a prevention strategy for chronic diseases and a useful tool for the healthcare system to reduce the administration of expensive and often not effective treatments. The chemical characterization of a methanolic extract (AJ) of *Ajuga reptans* L. was performed, and its antioxidant activity was evaluated. AJ and the major compounds, characterized by chromatographic techniques as phenylpropanoids and iridoids, were able to reduce the Reactive Oxygen Species levels in cancer cell lines (melanoma, A375, cervical cancer, HeLa, and alveolar adenocarcinoma, A549), stimulated by *E. coli* lipopolysaccharide. However, a clinical translation of these results encountered a significant limitation represented by the poor water solubility and bioavailability of the extract and compounds. Consequently, a hydro-soluble powder system (AJEP_3_) was developed by spray-drying encapsulating AJ into a multi-component solid matrix that is based on L-proline and hydroxyethylcellulose as loading and coating agents, and lecithin as solubility enhancer. The technological approach led to a satisfactory process yield (71.5%), encapsulation efficiency (99.9%), and stability. The in vitro water dissolution rate of the bioactive compounds appeared to be improved with respect to the extract, suggesting higher feasibility in the manufacturing and administration; even the in vitro biological activity of the produced multi-component AJEP_3_ was clearly enhanced.

## 1. Introduction

Reactive oxygen species (ROS), such as superoxide anion (O_2_^−^), hydrogen peroxide (H_2_O_2_), and hydroxyl radical (^•^OH), are produced during the oxygen metabolism and cellular respiration. Under normal conditions, the cell activates endogenous defence mechanisms (enzymatic and non-enzymatic) to counteract the potential damage of ROS. However, infection states (by bacteria, fungi, viruses), malfunction of mitochondria, or exposure to environmental factors increase the production and intracellular accumulation of free radicals, which, if not neutralized by the effective activation of the endogenous defense system, lead to oxidative stress [1]. The harmful effects of ROS are manifested by modifications of lipids of plasma membranes, proteins, and nucleic acids of DNA and RNA, resulting in mutagenicity, neoplastic transformation, or cell death. The imbalance between the overproduction of ROS/endogenous protective mechanisms, in favour of the former, is, therefore, the basis of various pathologies, not only carcinogenesis, but also inflammation, diabetes, neurodegenerative diseases, and cellular aging [2].

In these cases, an efficient strategy for reducing the damages of the oxidative stress, which negatively affects cell viability, is the administration of exogenous antioxidants, mainly obtained from natural sources, like herbs or functional foods, which show fewer side effects than synthetic molecules. The identification of extracts or isolated bioactive molecules from plants with antioxidant activity underlies the development of innovative nutraceutical and pharmaceutical formulations, which are useful for maintaining human well-being and the prevention or treatment of acute and chronic pathologies. In general, the biological effectiveness of plant extracts is related to the presence of secondary metabolites, such as polyphenols and terpenoids [3,4].

Polyphenols, including glycosylated phenylpropanoids, perform their antioxidant activity acting as direct scavenging of free radicals, as they stabilize radical molecules by donating hydrogen atoms or electrons [5]. The deriving phenolic radical is more stable because of the delocalization of the unpaired electron on the aromatic ring, and the chain of radical reactions is broken. Furthermore, the ability to chelate metal ions, such as iron, and enzymes, such as xanthine oxidase, cyclooxygenase, and lipoxygenase, which catalyze the formation of ROS, are other mechanisms that contribute to the antioxidant power of polyphenols [5].

*Ajuga* genus (Lamiaceae botany family) includes about 300 species widespread in the temperate regions of Europe, Asia, Australia, North America, and Africa [6,7,8]. In Italy, the perennial herbaceous *A. reptans* L. species, known with the common name of “bugola”, grows throughout the territory up to the mountain belt at 1500 m high. The folk medicine attributes to *A. reptans* L. anti-diabetic, anti-hypertensive [9], diuretic [10], and hepatoprotective properties [11]. Ecdysteroids, triterpenes, sterols, diterpenes, iridoids, and flavonoids have been identified as bioactive compounds providing antioxidant power, antimicrobial, and anti-inflammatory activity [6,11,12,13]. Moreover, *Ajuga* extracts and the major phenylpropanoids were demonstrated as both antioxidant and UV protector agents in cosmetic formulations to counteract premature cell ageing [14].

However, the practical application of plant extracts or herbal preparations in human health-promoting formulations presents issues that are related to the administration of substances with poor water solubility, bioavailability, and structural integrity [3]. The large-scale use of natural products is also limited by the low long-term stability to environmental factors, such as light, oxygen, and heat, which may induce oxidation or other chemical modifications with, sometimes, the loss of the biological activity. Many plant extracts have an unpleasant odour, an intense colour (dark green, brown), and an astringent and bitter taste that complicate their use in oral formulations [15].

These drawbacks may be overcome by the microencapsulation of plant derivatives into a polymeric matrix, which is able to protect them from the surrounding environment, mask the unwanted organoleptic characteristics, increase the solubility in water and biological fluids, and control the release towards a physiological target [16,17]. Among the physical encapsulation techniques, spray-drying is widely used in pharmaceutical, food, and cosmetic industries. It is a single-step process that transforms a solution/suspension, composed of loading and coating polymers and active ingredients, into a microparticulate powder through atomization in a heat-drying chamber [18,19]. This “mild” technology is suitable to encapsulate heat-sensitive materials, like plant derivatives, owing to the short exposure time of the feed droplets to the hot air into the drying chamber. Nevertheless, a challenge is represented in finding the balance between the physical-chemical characteristics of the materials to be encapsulated and the process parameters to optimize the particle production. One of the causes of unsatisfactory production yields may be the low glass transition temperature of many raw materials, and an imbalance in the composition of the feed matrix can worsen the process efficiency. As a result, the sprayed material may form a paste-like soft structure with high stickiness, affecting, not only process yield, but also the recovery and the stability of the particles. Appropriate additives, used in the right concentrations, may improve the formation process of microparticulate powders as well as their stability [20].

In a recently published work, a multi-component matrix that is based on L-proline/hydroxyethylcellulose as loading/coating agents, and lecithin as solubility enhancer, was developed to encapsulate, via spray-drying, an organic functional extract from hazelnut shells [21]. The matrix was able to well interact with the extract enhancing its stability, handling, and water dissolution rate resulting in a high-quality bioactive ingredient for natural healthy products [21]. We proposed this formulative and productive approach as a general model to carry and deliver extracts with similar physico-chemical characteristics and critical solubility and stability, giving a microparticulate product with extended shelf-life and improve technological particle properties, such as morphology, shape, and water dissolution rate.

Based on these considerations, in the present study, the quali-quantitative chemical characterization of an extract (AJ) from *Ajuga reptans* L. was carried out. The antioxidant effect of AJ and its major components on Reactive Oxygen Species levels in cancer cell lines (melanoma, A375, cervical cancer, HeLa, and alveolar adenocarcinoma, A549), stimulated by *E. coli* lipopolysaccharide, was evaluated. The final goal was to verify the possibility to load and carry AJ extract in the multi-component system based on L-proline/hydroxyethylcellulose/lecithin. The produced delivery system (AJEP_3_) was characterized in terms of yield (%) and encapsulation efficiency (%); particle size, morphology, water dissolution rate, and biological efficacy were evaluated with respect to raw extract as well as the stability under harsh storage conditions.

## 2. Materials and Methods

### 2.1. Chemicals and Reagents

*N*-hexane, chloroform (CHCl_3_), methanol (MeOH), glacial acetic acid (AcOH), and butanol (*n*-BuOH) of the analytical grade used for extraction and isolation procedures, deuterated methanol (CD_3_OD), the folin-ciocalteau reagent, the radical 1,1- diphenyl-2-picrilhydrazilic (DPPH), 2,2′-azino-bis (3-ethylbenzothiazolin)-6-sulfonic acid (ABTS), Trolox, MeOH HPLC-grade, formic acid (HCOOH), dimethyl sulfoxide (DMSO), gallic acid, α-tocopherol, BHT (buthylhydroxyaniosole), potassium persulfate (K_2_S_2_O_8_), sodium chloride (NaCl), anhydrous dibasic sodium phosphate (Na₂HPO₄), anhydrous monobasic sodium phosphate (NaH_2_PO_4_), and L-proline (P) were provided by Sigma–Aldrich (Milan, Italy). A Milli-Q50 filtration system (Millipore Corp., Bedford, MA, USA) was employed to prepare the HPLC-grade (18 mΩ) water. For the electrospray ionization ESI-MS analysis was used water and MeOH of HPLC supergradient quality (Romil Ltd., Cambridge, UK). Lecithin (L, E322) and medium viscosity hydroxyethylcellulose (HEC, Natrosol MR) were purchased from ACEF Spa (Fiorenzuola D’Arda, Italy). Human cancer cell lines of melanoma (A375), alveolar adenocarcinoma (A549), and cervical carcinoma (HeLa) were purchased from ATCC. Gibco Life Technology Corp. (Thermo Fischer Scientific, Weil am Rhein, Germany) has provided reagents and supplements for both cell culture and antioxidant activity evaluation.

### 2.2. General Experimental Procedures

For NMR spectra, a Bruker DRX 600 (Bruker BioSpin GmBH, Rheistetten, Germany) NMR spectrometer was used, operating at 599.2 MHz for ^1^H and 150.9 for ^13^C, while using the TopSpin 3.2 software package. The chemical shifts (expressed in δ, parts per million) at δ_H_ 3.31 and δ_C_ 49.05 of the deuterated CD_3_OD, were used as a reference, and the coupling constants, *J*, were expressed in Hertz. For ^1^H–^1^H DQF-COSY, ^1^H–^13^C HSQC, and HMBC experiments were used conventional pulse sequences [22].

For thin-layer chromatography (TLC), precoated Macherey−Nagel, type Kiesegel 60 F_254_, 0.25 mm thick plates (Milan, Italy), and Ce((SO_4_)/H_2_SO_4_ and UV (254 and 366 nm) were used for compound visualization. Sephadex LH-20 (dextran) from Pharmacia (Pharmacia, Uppsala, Sweden) was used as a stationary phase for column chromatography with molecular exclusion. The semi-preparative separations for HPLC were carried out with a Waters device, consisting of a Waters 590 series pumping system, equipped with a Waters R401 refractive index detector, a Rheodyne injector (100 µL loop), using a C_18_ µ-Bondapack (300 × 7.8 mm particle size 10 µm, Waters, Corp., Milford, MA, USA) as a column. The quantitative HPLC analysis was carried out with an Agilent 1100 Series system (Millipore, Boston, MA, USA), connected to a binary pump mod. G-1312, Rheodyne injector mod. 1328A (20 µL loop), a degasser mod. G-1322A, and a photodiode detector G-1315A. The peak area was calculated using an Agilent supplement.

### 2.3. Plant Material and Extract Preparation

The whole plant of *A. reptans* L. (Lamiaceae) was purchased in a plant nursery (De Santis, Baronissi, Salerno, Italy), in June 2018. A voucher specimen (AJ0618) is deposited at Pharmacy Department of University of Salerno. The aerial parts and roots were separately dried in an oven (Kendro Laboratory Products Heraeus Microbiological Incubator D-63450, Hanau, Germany), at 37 °C, for six days and then ground with Grindomix (mod. RM 100, Retsch, Bergamo, Italy) at 4000 rpm for four minutes.

Briefly, dried and ground whole plant (flowers, aerial parts, and roots) (200 g) was initially defatted with *n*-hexane (3 times × 2 L for 24 h) and chloroform (3 times × 2 L for 24 h) and then extracted, at room temperature, with methanol (3 times × 2 L for 24 h) [22]. The extraction solvent was evaporated under vacuum at 40 °C in a rotary evaporator (B690, BUCHI Italia s.r.l, Milan, Italy) in order to obtain the dried residue (AJ, 48.7 g).

The extraction yield (24.3% *w*/*w*), determined gravimetrically (Precisa Gravimetrics AG, Dietikon, Switzerland, max 410 gd = 0.1 g; +15/30 °C), was calculated as:

Extraction yield (%) = (weight of dry extract (g)/weight of the initial powder (g)) × 100.

### 2.4. Isolation Procedures and Chemical Characterization of AJ Compounds

A portion of the methanolic extract (6.63 g) was suspended in water and then fractionated with *n*-BuOH, used as a solvent with high affinity for polyphenols to obtain the n-BuOH extract enriched in polyphenols compounds [23,24]. An extract aliquot (3.1 g) was fractionated by molecular exclusion chromatography on a Sephadex LH-20 column (1 m × 3 cm i.d.) while using MeOH as eluent (flow rate, 1 mL/min.). The fractions (6.0 mL each) were collected and checked by TLC (Si-gel, *n*-BuOH-AcOH-H_2_O 60:15:25, CHCl_3_-MeOH-H_2_O 7:3:0.3). Fractions with similar Rf values were collected into III main groups and then separated by RP-HPLC chromatography. The fraction I was separated using a mixture of MeOH-H_2_O 25:75 *v*/*v* as the mobile phase, a flow rate of 2.0 mL/min., and a µ-Bondapack C_18_ (Waters) as a column. From the fraction I (1440 mg) harpagide (1) (32.0 mg, t_R_ 5.0 min.), 8-O-acetylharpagide (2) (33.0 mg, t_R_ 12.0 min.), and reptoside (3) (9.5 mg, t_R_ 25.0 min.) were identified as pure compounds. Fraction II (445.8 mg) was purified using a MeOH-H_2_O (40:60) *v*/*v* (flow rate of 2.0 mL/min.) and a µ-Bondapack C_18_ (Waters) as a column isolating teupolioside (4) (10.7 mg, t_R_ 12.0 min.) and martinoside (5) (15.5 mg, t_R_ 44.0 min.). Fraction III (175.0 mg) was separated using MeOH-H_2_O 35:65 *v*/*v*, a flow rate of 2.0 mL /min., and a µ-Bondapack C_18_ (Waters) as a column, provided the compound (**6**) verbascoside (15.6 mg, t_R_ 20.0 min.) and the compound (**7**) isoverbascoside (2.3 mg, t_R_ 35.0 min.).

The structural elucidation of the compounds that were isolated from AJ was obtained through the combined use of mono (1H, ^13^C, and TOCSY) and two-dimensional (HSQC, HMBC, and DQF-COSY) NMR techniques and mass spectrometry (ESIMS), in comparison with the data reported in the literature [25,26,27,28].

### 2.5. Quantitative Determination of Total Phenol Content

The Total Phenolic Content (TPC) of AJ was determined using the Folin–Ciocalteu colourimetric assay, following the previously reported method [29]. The results were expressed as gallic acid equivalents (GAE) (y = 0.0066x + 0.0507, GAE mg/100 g of extract, means ± standard deviation of three determinations).

### 2.6. Quantitative HPLC Analysis

The quantitative analysis was carried out by HPLC-DAD using a μ-Bondapak C_18_ column (300 × 3.9 mm, 10 μm, Waters), flow 1.0 mL/min., injection volume 20 μL. The elution was performed on a gradient. The solvents used were H_2_O + HCOOH 0.1% (A) and MeOH + HCOOH 0.1% (B). The selected elution gradient was 0→5 min, 20% B; 5→10 min, 20→30% B; 10→20 min, 30% B; 20→30 min, 30→35% B; 30→35 min, 35% B; 35→45 min, 35→40% B; 45→55 min, 40→100% B. Absorbance was monitored with a UV-DAD detector, model G-1315A, set at λ 330 nm. The attribution of the chromatographic peaks was deduced from the retention time of each compound and then confirmed by co-injection with the standards of the isolated compounds. Teupolioside (**4**), martinoside (**5**), and verbascoside (**6**) were identified as the main compounds of AJ. The extract was analyzed at the concentration of 30 mg/mL; the pure compounds (**4**–**6**) were analyzed in a range of concentrations 0.5–2.0 mg/mL, in order to produce calibration curves. The standard curves of compounds **4**–**6** were analyzed using the linear correlation between the concentration and the peak area (regression equation y = 9037.1x + 1317.8, R^2^ = 0.9996, for compound **4**, y = 14509x − 65.27, R^2^ = 0.9993 for compound **5**, and y = 6337.1x − 159.07, R^2^ = 0.9997, for compound **6**, where y is the peak area and x the compound concentration).

### 2.7. Chemical-Based Free Radical Scavenging Activity

#### 2.7.1. Diphenyl-2-Picrylhydrazyl Radical (DPPH) Test

The free radical scavenging activities of the AJ extract and the main compound (teupolioside, **4**) were investigated against 1,1-diphenyl-2-picrylhidrazyl (DPPH). The used concentration range was from 2.5 to 400 μg/mL, according to [30]. A calibration curve (y = 0.0228x − 0.0350, R^2^ = 0.9999) of DPPH (concentration range = 5–36 µg/mL) was applied to determine the concentration of DPPH in the reaction medium. The equation: % RSA = 100 (A_0_ − As)/A_0_ (A_0_ = absorbance of a control solution without extract/compound and A_S_ = absorbance of the solution with the extract or compound) was used to calculate the radical scavenging activity (RSA) of the extract or compound.

The EC_50_ (average effective scavenger concentration) was determined as the amount of the sample (in micrograms per millilitre) necessary to decrease the initial concentration of DPPH by 50%. All of the tests were performed thrice. Low EC_50_ value indicates a higher free radical scavenging activity.

#### 2.7.2. Trolox Equivalent Antioxidant Capacity (TEAC) Assay

The generation of the radical cation of ABTS (2,2′-azino-bis (3-ethylbenzothiazolin)-6-sulfonic acid) and all other steps of the test was performed, as reported in [31], using as concentrations 0.0205 to 0.082 mg/mL for AJ and 0.00075–0.05 mM for pure compound (**4**). The percentage of scavengers was calculated with respect to Trolox (a water-soluble analogue of vitamin E used as a positive test control). The antioxidant activity was expressed as equivalent of Trolox (TE) mM/mg of extracts or mM of compounds. High TEAC value indicates a higher free radical scavenging activity.

### 2.8. Antioxidant Activity in Cell Systems

#### 2.8.1. Cell Culture

The cell lines of human malignant melanoma (A375), human cervical carcinoma (HeLa), and alveolar adenocarcinoma (A549) were grown in Dulbecco’s modified Eagle’s medium containing high glucose supplemented with 10% fetal bovine serum, 100 U/mL each of penicillin and streptomycin, and 2 mM/L glutamine, and they were then kept at 37 °C under 5% CO_2_ humidified air.

#### 2.8.2. Cell Viability

The potential cytotoxic effect of AJ extract, compounds **1**–**6**, and AJEP_3_ microparticles, was investigated by MTT (3-(4,5-dimetiltiazol-2,5-diphenyl-2H-tetrazolium bromide)) assay, as reported in [21]. Approximately, cells (5.0 × 10^4^/well) were grown in 96-well plates and, after 24 h, were treated with extract or compounds, dissolved in DMSO, and AJEP_3_ microparticles dissolved in distilled water. Subsequently, after a short spin at 1200 rpm, the medium was removed and 100 μL of 1 mg/mL MTT was added to each well. The plates were incubated at 37 °C for 2 h to allow the formation of formazan salt. Next, the salt crystals of formazan were dissolved with 100 μL of DMSO. Absorbance (620 nm) for each point was calculated by a Thermo Scientific Multiskan Spectrum (ThermoFisher Scientific, Weil am Rhein, Germany). A mortality of less than 50% was considered to be non-cytotoxic.

#### 2.8.3. Cell Treatment

A375, HeLa, and A549 were plated on multiwell plates, and serial dilutions of AJ (200−100−50 µg/mL), compounds (75−50−25 µg/mL), AJEP_3_ microparticles (0.5−1.0−1.5 mg/mL) were added for one hour, and then co-exposed to lipopolysaccharide (LPS), from *Escherichia coli*, (0.1 µg/mL) for 24 h in order to evaluate the antioxidant activity of extract (AJ), isolated compounds (**1**–**6**), and AJEP_3_ microparticles [32].

#### 2.8.4. Antioxidant Activity

The antioxidant activity of AJ, compounds, and AJEP_3_ microparticles was investigated using a cytofluorimetric assay, 2′–7′-dichlorofluorescein diacetate (H_2_DCF-DA), usually adopted to evaluate the intracellular production of peroxides [32]. H_2_DCF-DA is a non-polar molecule that easily diffuses inside cells and it is hydrolysed by intracellular esterases that remove the acetate groups making it polar and therefore impermeable. H_2_DCF-DA is quickly converted to a fluorescent product, DCF, which may be analyzed using a flow cytometer (the excitation wavelength is 504 nm and the emission wavelength is 529 nm).

Briefly, A375, HeLa, and A549 cells (2.0 × 10^4^/well) were seeded into 12-well plates and then treated, as described previously. After collecting the cells and washing with PBS buffer, a solution containing H_2_DCF-DA (10 µM) was added at 37 °C. After 45 min. of incubation, cells’ fluorescence was rated using a fluorescence-activated cell sorting and analyzed with Cell Quest software [32]. The results are reported by mean fluorescence intensity.

### 2.9. Microencapsulation Process

#### Polymeric Matrix Preparation and Spray Drying Conditions

The polymeric matrix was chosen based on a previously conducted study [21]; different liquid feeds were prepared following the Hot-Cold (H-C) method that was reported by Esposito et al., and briefly described here.

5% *w*/*v* of L-proline (P) and 0.2% *w*/*v* of hydroxyethylcellulose (HEC) alone, or in combination with lecithin (L), at 0.2% *w*/*v* or with a mixture of aqueous ethanol 80:20 as a solvent system were used.

In the Hot-Cold method (H-C), water was added to HEC to obtain the aqueous HEC solution, heated to 75 °C, and P added at room temperature, in a 98:2 P:HEC ratio (total amount 5.20 g). The matrix was added to AJ amount in the range between 0.50–0.60% *w*/*v* of AJ, sonicated 30 min, and then left under stirring until the spray drying process to obtain loaded microparticles (AJEP_1_).

The Hot-Cold method was slightly modified when lecithin (L) was added to the feed, as follows: P was dissolved in 20 mL of 1% *w*/*v* L solution, added in the range between 0.50–0.60% *w*/*v* of AJ, and then mixed with a previously prepared HEC solution (0.2 g in 80 mL of water). The liquid feed was sonicated 30 min. before to be sprayed obtaining AJEP_2_ microparticles.

In the final optimized liquid feed preparation, 20 mL of ethanol was mixed with 20 mL of a 1% *w*/*v* L solution and added to AJ extract (0.55% *w*/*v*). The obtained suspension was slowly poured into the aqueous P-HEC feed, under continuous magnetic stirring until being processed, to obtain loaded microparticles (AJEP_3_).

Unloaded batches (blank powders) were prepared in the same condition, without AJ extract.

All of the liquid feeds were spray-dried by a Mini Spray Dryer Büchi B-290 (Büchi Laboratoriums-Technik, Flawil, Switzerland) equipped with a Ø nozzle = 0.7 mm under the following experimental conditions: air pressure 6 bar, drying airflow 600 L/h, inlet/outlet temperatures 125/76 °C, aspirator 100%, and spray flow feed rate of 3.3 mL/min.

Each batch was produced in triplicate. All of the powders were stored in the desiccator (72 h) before further characterizations.

### 2.10. Powders Characterization

#### 2.10.1. Yield and Loading Efficacy

The production yield was calculated by weighing the collected sample (Crystal 100 CAL balance max 110 gd = 0, 1 mg; +15 °C/30 °C, Gibertini Novate, Milanese, Italy) and then expressed as the percentage of the final product to the total quantity of materials sprayed.

The Actual (AEC) and Theoretical (TEC) Extract Content and their ratio for determining the Encapsulation Efficiency (EE%) were calculated, as reported by Esposito et al., 2017 [22], by the following equation:

EE% = AEC/TEC × 100(1)

The results have expressed as the average value obtained by the triplicate of the analysis.

#### 2.10.2. Quantitative Analysis

##### HPLC Method

The teupolioside (**4**) content in AJEP_3_ was evaluated by HPLC, using the experimental condition reported in paragraph 2.6., dissolving the sample directly in water, at 30 mg/mL.

##### UV Method

The teupolioside (**4**) content was also investigated by UV measurement (UV/Vis spectrometer Lambda 25, Perkin Elmer Instruments, Waltham, MA, USA) at λ 327 nm. The compound was dissolved in water in a range between 2.5–200 mg/L, and the teupolioside calibration curve was generated by the mean absorbance as a function of concentrations (y = 0.0031x + 0.0014, R^2^ > 0.9999).

#### 2.10.3. Morphology and Particle size Distribution

The morphology and mean size of raw materials and spray-dried powders, coated with Au/Pd, were investigated by scanning electron microscopy (SEM) (Carl Zeiss EVO MA 10 microscope operating at 17 kV). The average particle diameters were determined from an average of at least 20 observations.

Fluorescence (FM) analyses were carried out using a fluorescence mode, a standard DAPI (40,6-diamidino-2-phenylindole) optics adsorbing violet radiation (λ 372 nm) and emitting a blue fluorescence (λ 456 nm) with a Zeiss Axiophot microscope (Carl Zeiss Vision, München-Hallbergmoos, Germany) in optical mode, equipped with 63 and 100 × 1.4 NA plan Apochromat oil and non-oil immersion objectives. Before undergoing FM analysis, the powder samples were housed on the glass slide, distributed using airflow, and then dispersed in petroleum jelly oil.

The size and size distribution of either spray-dried particles and raw materials were determined by Laser Light Scattering (LLS) granulometry (Beckman Coulter LS 230, Particle Volume Module Plus, Brea, CA, USA).

All of the samples were suspended in isopropanol. The analysis was conducted introducing 150 μL of each sample into the cell to obtain an obscuration between 8% and 12%. The tests were triplicated. The mathematical Fraunhofer model was applied to calculate the particle size distribution and d_50_, the volume diameter at the 50th percentile of the particle size distribution, was used to report the results.

The span value was determined as ((d_90_ − d_10_)/d_50_); a span value >3 was correlated to a non-homogeneous particle size distribution [33].

#### 2.10.4. Dissolution/Release Test

In vitro dissolution/release tests of AJ and AJEP_3_ powders were carried out, according to the Farmacopea Ufficiale Italiana [34].

AJ solubility was calculated preparing a saturated water solution (8 mL) of extract into glass vials (stirred and stored at 25 °C for 3 days). Subsequently, the samples were subject to centrifugation (at 3000 rpm for 15 min.) and the supernatants were recovered through filtration (0.45 µm). The UV method described before (paragraph 2.10.2) was employed in order to determine the concentration of dissolved AJ evaluated as teupolioside (**4**) (the water-soluble chemical marker of AJ) equivalents. The measurements were performed in triplicate.

Based on the determined AJ water solubility (115.30 ± 0.27 mg/L), the sink conditions for both dissolution/release tests of the unprocessed extract AJ (40 mg/L) and spray-dried formulations (100 mg/L, when considering the extract content) were calculated.

For dissolution tests, carried out by a Sotax AT Smart Apparatus (Basel, Switzerland), paddle 75 rpm at 37 °C, equipped with apparatus 2 of USP 31, 100 mg of AJEP_3_ and 40 mg of AJ were dissolved in 1000 mL of distilled water (sink conditions).

The amount of dissolved extract was measured as AAC (Actual Active Content) value, calculated by the UV method reported in the previous paragraph (Section 2.10.2). The dissolution/release test (60 min. with samples taken each 5 min.) was performed 18 times during three different tests each one in six vessels (three times for six vessels), and the mean values were reported (standard deviations <5%).

#### 2.10.5. Differential Scanning Calorimetry (DSC)

The thermal behaviours of all materials were analysed by Differential Scanning Calorimetry on indium calibrated Mettler Toledo DSC 822e (Mettler Toledo, Worthington, OH, USA); 3–6 mg of each sample was weighed with a microbalance MTS Mettler Toledo (Worthington, OH, USA) in a 40 μL accurately sealed and pierced aluminium pan. All of the samples underwent one dynamic thermal cycle; they were heated, at a heating rate of 10 °C/min., from 25 to 350 °C.

### 2.11. Study of AJ Chemical Stability and AJEP_3_ Functional Activity

**Figure 1:**
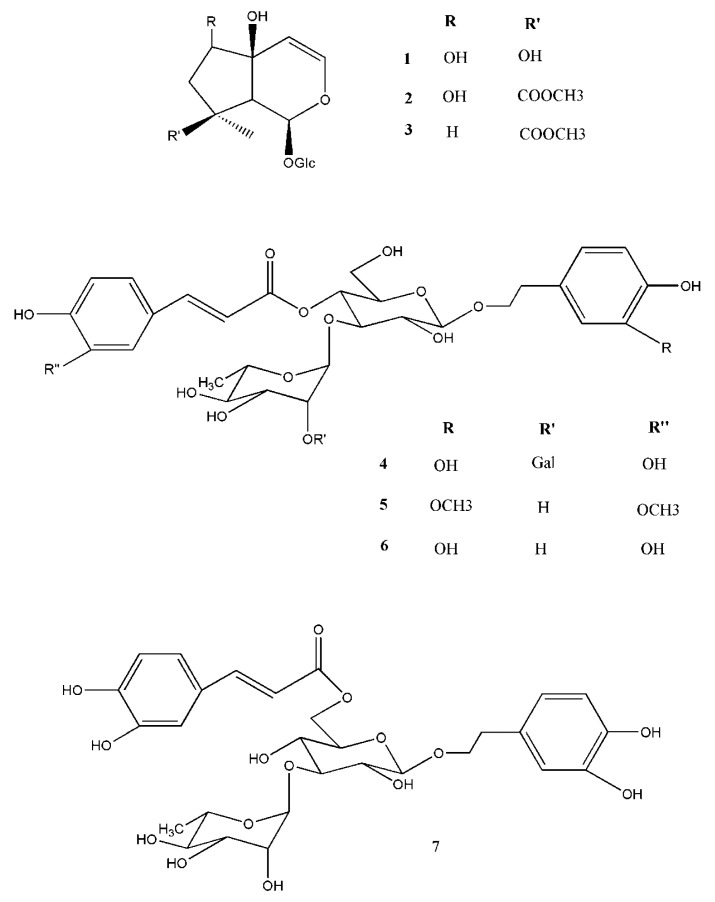
Major chemical constituents of AJ extract: iridoid glycosides (harpagide (**1**), 8-O-acetylharpagide (**2**), and reptoside (**3**)) and phenylpropanoid glycosides (teupolioside (**4**), martinoside (**5**), verbascoside (**6**), and isoverbascoside (**7**)).

Tapped glass vials containing 1 g of sample (AJ or AJEP_3_) were stored in a climatic chamber (Climatic and Thermostatic Chamber, Mod. CCP37, AMT Srl, Milan, Italy) for six months at 40 °C ± 2 °C with 75% RH ± 5% (accelerated stability test, ICH guidelines) [35]. At given times (0 and after six months) samples of each batch were collected. The *t*_0_ corresponds to 72 h from the formulation. Teupolioside (Figure 1, structure 4) content was verified by the previously described HPLC method (Section 2.6).

The free radical scavenging of AJEP_3_ was performed by chemical-based tests against DPPH^•^ and ABTS^•^^+^ radicals, dissolving AJEP_3_ (from 1000 to 200 µg/mL), in distilled water, using the procedure already reported for AJ in Section 2.7.

Additionally, the in vitro antioxidant activity of AJEP_3_ in human malignant cells, A375, HeLa, and A549 was determined. AJEP _3_ was directly dissolved in the culture medium, at 0.5–1.0–1.5 mg/mL, and the test was carried out in triplicate applying the method and experimental conditions reported in Section 2.8. The obtained results for AJEP_3_ were compared with those of the raw extract (AJ).

### 2.12. Statistical Analysis

The results of the experimental plan are reported as mean ± standard deviation of three replications for each test, performed in triplicate. Statistical analysis between data groups was carried out while using ANOVA followed by the Bonferroni parametric test or Tukey HSD test using the software GraphPad Prism version 7.00 for Windows. Differences were considered to be significant if *p* < 0.05.

## 3. Results and Discussion

### 3.1. Ajuga reptans L. Extract Preparation, Chemical Composition, and Quantitative Analysis

The chemical characterization of a plant extract with potential applications in human health-promoting products ensures the reproducibility of the extraction method and the standardization of biological effects.

AJ was subjected to molecular exclusion chromatography and RP-HPLC obtaining seven major constituents to identify the main secondary metabolites. The structures of the isolated compounds (Figure 1) were elucidated by their NMR and MS data in comparison with those being in literature. AJ constituents include three iridoid glycosides (**1**–**3**), harpagide (**1**) [25] 8-O-acetylharpagide (**2**) [25], reptoside (**3**) [25], and four phenylpropanoid glycosides (**4**–**7**), teupolioside (**4**) [26], martinoside (**5**) [27], verbascoside (**6**) [36], and isoverbascoside (**7**) [28].

Monoterpenoids (**1**–**3**) and polyphenols (**4**–**7**) are characteristic secondary metabolites of the *Ajuga* genus [11,12], exerting many health properties, having antioxidant, anti-inflammatory, and antibacterial effects [7,13].

The quantitative analysis that was performed by RP-HPLC-DAD showed that the phenylpropanoids, teupolioside (**4**), verbascoside (**6**), and martinoside (**5**) are the main constituents, representing 2.8 ± 0.4, 0.9 ± 0.2, and 0.7 ± 0.3%, *w*/*w*, respectively, of the dried extract with teupolioside (**4**) as the chemical marker of the extract.

### 3.2. Chemical-Based Free Radical Scavenging Activity

Despite few iridoid glycosides showing antioxidant properties [37], glycosylated phenylpropanoids are powerful antioxidants acting by scavenging reactive oxygen and nitrogen species, chain-breaking peroxyl radical, or chelating metal ions [38].

Verbascoside also inhibits LDL peroxidation in vitro, protects endothelial cells from oxidative stress induced by free radicals, and liver cells from toxicity induced by carbon tetrachloride [39,40]. Additionally, teupolioside (**4**) has demonstrated powerful anti-inflammatory, antioxidant, and chelating properties of bivalent ions [38].

For this reason, AJ, rich in phenypropanoids, was tested to evaluate its free radical scavenging activity towards the two stable radicals, DPPH^•^ and ABTS^•+^, frequently employed to estimate antioxidant capacities in plant derivatives. The results showed that AJ exhibited a significant and dose-dependent free radical scavenging activity against both DPPH^•^ (EC_50_ 135.78 ± 4.12 µg/mL) and ABTS^•+^ (0.29 ± 0.01 mM Trolox/mg extract) as compared to the positive test controls (α-tocopherol EC_50_ 10.10 ± 1.30 µg/mL, and BHT 0.36 ± 0.03 mM Trolox/mM compound). AJ activity is related to the occurrence of a high amount of total polyphenols (135.78 ± 4.12 mg_GAE_/100g of AJ) [22,30,31], such as phenylpropanoids [38,41,42], such as teupolioside (**4**) (EC_50_ 9.18 ± 4.12 µg/mL, and TEAC value of 0.64 ± 0.04 mM Trolox/mM compound).

### 3.3. Antioxidant Activity in Cell Systems

Testing the in vitro antioxidant activity in cellular systems is crucial for considering the critical aspects of the real in vivo effect, like bioavailability, uptake, and ability of the sample to cross plasma membranes [43]. Cancer cell lines have an overproduction of ROS with respect to the normal cells, and, for this reason, employed to assess the assay [44,45]. The use of the redox probe dichlorofluorescein diacetate (H_2_DCF-DA) is a common tool for screening the antioxidant properties of natural derivatives. H_2_DCF-DA easily oxides to fluorescent form (DCF) in the presence of a free radical in the cellular medium. The ability of an antioxidant extract or compound to reduce the fluorescent signal, detectable, and quantifiable by flow cytometer, is directly correlated to its antiradical activity [43].

Based on the positive preliminary chemical results, the effect of AJ extract and the main isolated compounds (**1**–**6**) on the level of *Escherichia coli* lipopolysaccharide-induced Reactive Oxygen Species (ROS), in human tumour cell lines (melanoma A375, cervical cancer HeLa, and alveolar adenocarcinoma A549) was evaluated at non-cytotoxic concentrations (AJ up to 200 µg/mL and compounds up to 75 µg/mL). Compound **7** (isoverbascoside) was not tested due to the low isolated amount (see the experimental part, Section 2.4).

Human tumour cell lines exhibit the simultaneous activation of multiple cell surface receptors and signalling pathways hence targeting one node typically leads to the activation of alternative pathways. The development of novel compounds with low toxicity and improved efficacy is of great interest.

**Figure 2:**
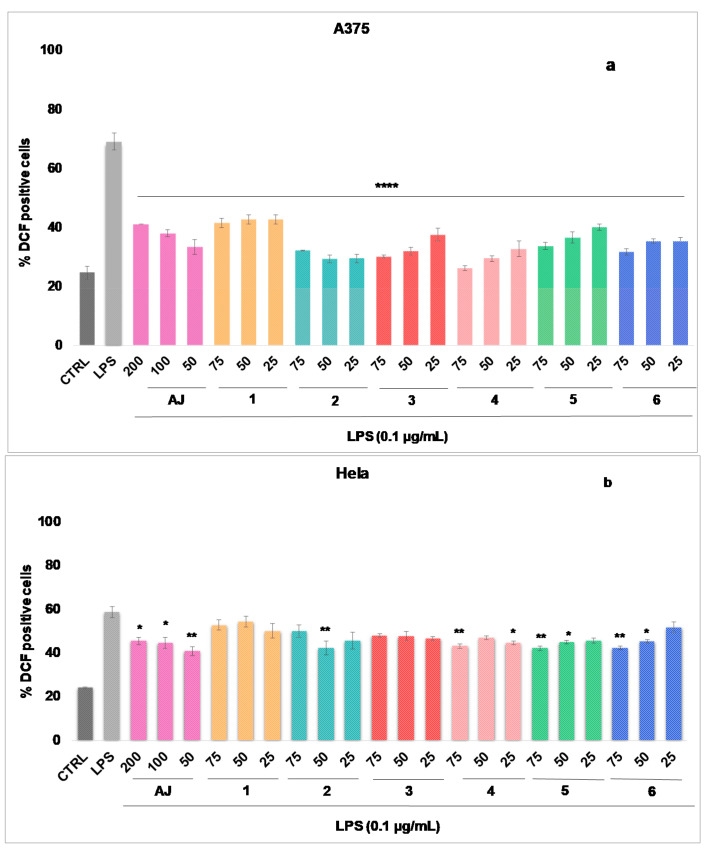

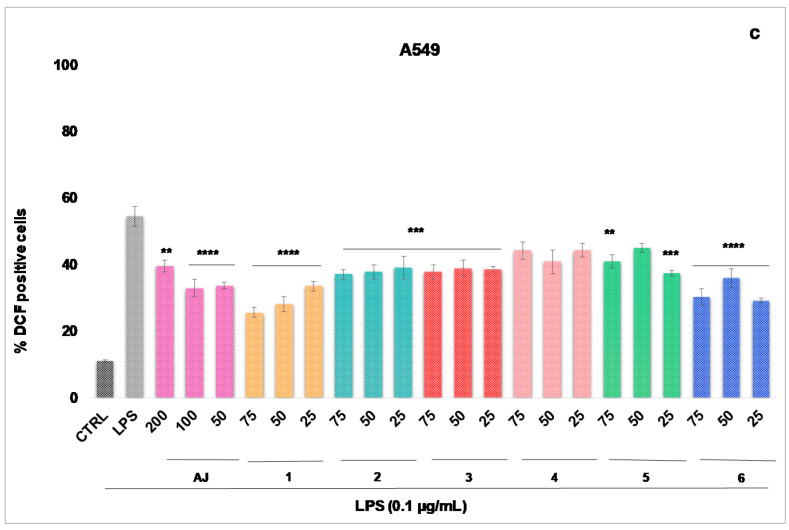
Reactive oxygen species (ROS) formation was evaluated through the probe 2′,7′ dichlorofluorescein diacetate (H_2_DCF-DA) in A375 (**a**), HeLa (**b**), and A549 (**c**) cells. AJ (200–50 µg/mL) and pure compounds (**1**–**6**) (75–25 µg/mL) were added for 1h and then for further 24 h exposed to lipopolysaccharides from *Escherichia coli* (LPS; 0.1 μg/mL). ROS production was expressed as mean ± standard deviation of the percentage of 2′-7′-dichlorofluorescein (DCF) positive cells of at least three independent experiments each performed in triplicate. Data were analyzed by Student’s *t*-test. * *p* < 0.05, ** *p* < 0.005, and *** *p* < 0.001 vs. LPS at the same experimental conditions.

The results (Figure 2a–c) show that AJ significantly reduces the ROS level in the selected cell lines, showing a percentage of ROS reduction between 17.77 and 35.78%, starting from the concentration of 50 µg/mL. The inhibitory effect is more evident in A375 and A549 cells (% inhibition 35.78 and 30.33, respectively).

The antioxidant efficacy of AJ seems to be related to its chemical composition. Isolated and tested phenylpropanoids (**4**–**6**) also significantly inhibit (starting from 25 µg/mL) the intracellular ROS concentration, in the same cell lines. In A375 cells (Figure 2a), at 50 µg/mL teupolioside (**4**) (% inhibition of 39.62, *p* < 0.001) is more effective than verbascoside (**6**) (% inhibition of 33.73, *p* < 0.001), and martinoside (**5**) (% of inhibition of 32.58, *p* < 0.001). Among the iridoids, at 50 µg/mL 8-O-acetylarpagide (**2**) (% inhibition of 39.77, *p* < 0.001) is more active when compared to harpagide (**1**), and reptoside (**3**) (with% inhibition, respectively, of 29.50 and 37.13, *p* < 0.001). Verbascoside (**6**) (% inhibition of 28.12) and harpagide (**1**) (% inhibition of 35.82) among phenylpropanoids and iridoids, respectively, have a greater inhibitory effect on ROS production in cells A549 (Figure 2c).

Although several papers report the powerful antioxidant properties of phenylpropanoids in cell-free systems, few papers have shown their ability to inhibit the intracellular ROS levels, for example, in prostate cancer (PC3) or in colon carcinoma cells (HT-29) [37,39]. Therefore, the obtained results can contribute to a greater knowledge of the real counteracting the ROS cytotoxicity when the uptake of these molecules occurs in cell systems.

### 3.4. Microencapsulation Process

#### 3.4.1. Process Efficiency

The selection of an adequate polymeric matrix, liquid feed composition (viscosity, particle size, concentration), and the process parameters (flow rate, temperature) represents a crucial step to transform a natural extract in a stable powder, employing spray-drying technology. Usually, natural extracts are not soluble in water and they resulted in sticky material for the presence of low molecular weight sugar and acids, which have a low glass transition temperature [21].

In previous work, it has been shown how an amino acid, L-proline (P), with high intrinsic functional properties, can constitute a valid loading carrier for such extracts. Although both the extract and the amino acid had a high crystalline degree, an adequate amount a coating polymer as hydroxyethylcellulose (HEC) allows for the formation of a stable and easy-to-use microparticulate powder [21]. Between cellulose derivatives used as coating polymers in the microencapsulation process, hydroxyethylcellulose, a non-toxic polymer with hydroxyethyl groups along the polysaccharide chain, has right viscosity to control the release of the active embedded extract. L-proline, with high solubility in water and alcohol, has many biological functions, is involved in the biosynthesis of collagen, and protects the cell from oxidative stress. In the microencapsulation process, L-proline, like other amino acids, can be used as an enhancer of dispersion and loading polymer for active ingredients, mainly extracts. Lecithin (L), a natural emulsifier and food additive (E322), due to its amphiphilic character, is often used to increase the dispersion into the liquid feed of complex ingredients, such as plant extracts containing molecules with different polarity.

Preliminary experiments have been performed to verify the suitability of the multi-components matrix in loading and carrying AJ extract. Starting from the previous study, different weight ratios of P:HEC and the solvent system [water, ethanol (EtOH), and their mixtures] have been investigated to obtain an efficient process yield and to load the largest amount of AJ extract. Table 1 reports the most relevant results.

The best results were obtained using P:HEC ratio 98:2 in a 80/20 lecithin-water/EtOH solvent system and reported efficiency in Table 1 in terms of process yield, extract content, and encapsulation. The process yield, obtained for the powders deriving from the liquid feed without lecithin and ethanol, was not higher than 41%, probably because the poor water solubility of the extract negatively affecting the process, as shown in Table 1. The introduction of a small amount of ethanol in the liquid feed gave a process yield (AJEP_3_ Table 1) close to 72%. A process yield greater than 50% is an index of an efficient and scalable spray drying process; thus, the recovery of 71.50% obtained for AJEP_3_ was very satisfying [46]. The teupolioside content in AJ raw extract was 2.80 ± 0.5%. The same marker percentage with respect to the extract loaded (9.7% of total material processed), was recovered in AJEP_3_ microparticles. Thus, the encapsulation efficiency (EE%) resulted in being close to the 100% (99.9%, Table 1), confirming an efficient loading process with a total extract recovery.

#### 3.4.2. Powder Characterization

Information on AJEP_3_ powders derived from morphology and mean size analysis (SEM and LLS), extract-polymer physical interactions (DSC) were monitored in order to evaluate the solid-state, stability, actual active content, and free-radical scavenging activity of the spray-dried systems, before and after the microencapsulation process and during the storage period.

##### Solid-State, Morphology, and Particle size

The solid-state characterization (morphology, particle size, and particle size distribution, thermal analysis) of all processed and unprocessed powders was carried out.

**Figure 3:**
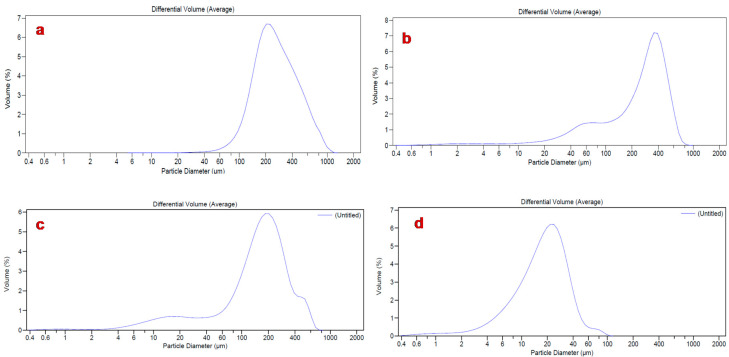
Size distribution analysis of L-proline (**a**), hydroxyethylcellulose (HEC) (**b**), AJ raw (**c**), and loaded powder (AJEP_3_, **d**).

As is well known, the shape of particles and reduced mean size are fundamental characteristics affecting the derived properties of powders that are relevant in the manufacturing industry. Particles with low mean size enhance their surface area exposed to the solvent, improving the dissolution rate and, consequently, the in vivo absorption [47]. The results obtained by Laser Light Scattering (LLS) analysis showed high particle size for all raw materials, with d_50_ values ranging from 277.22 µm for HEC and 250.10 µm for L-proline to 165.1 µm for AJ (Table 1, Figure 3a–c).

A reduced particle dimension was observed for the spray-dried systems EP_3_ (d_50_ 102.93 µm) and AJEP_3_ (d_50_ 18.46 µm) (Figure 3d, Table 1). This result could be due to the positive effect of both the spray-drying process and the balanced matrix ratio. Moreover, the markedly lowest value that was obtained for AJEP_3_ (d_50_ 18.46 µm, Table 1, Figure 3d) suggests that the presence of the extract promotes the physical interaction during the agglomeration process in forming smaller particles.

**Figure 4:**
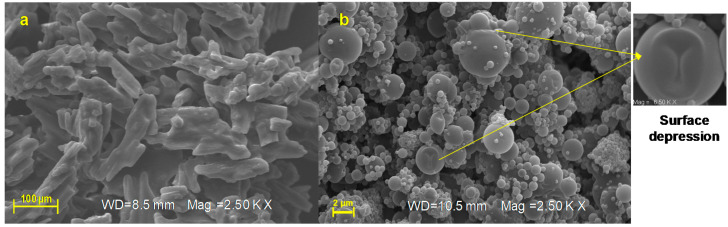
Scanning electron microscopy (SEM) micrographs (magnification: 2.5 KX, frame average, *n* = 1) of raw extract (AJ, (**a**)) and loaded powder (AJEP_3_, (**b**)) at t_0._

By morphological characterization, unprocessed AJ appears as a crystalline material (Figure 4a) made up of elongated and jagged crystals; otherwise, the developed technological approach by spray-drying was able to convert the crystalline form of starting extract (AJ, Figure 4a) in microsystems that consist of amorphous materials (AJEP_3_, Figure 4b).

SEM analysis indicated that AJEP_3_ is made of microparticles with almost spherical shape (Figure 4b) and low particle size (2–5 µm), but, as is visible by images in Figure 4b, the particles tend to form aggregates, leading to the highest dimensional distribution (Figure 3d). Anyway, the dimensional distribution resulted in being homogenous because microparticles showed a monomodal distribution curve with a low span value (1.63, Table 1).

Partial collapses of the particle surface that favour the cohesion between the particles as small joints are visible in Figure 4b and may be due to a depression of the internal volume during the solvent evaporation phase. This behavior is frequent when polymers form highly viscous liquid feed, which reduces the solvent evaporation rate during the spray-drying process.

**Figure 5:**
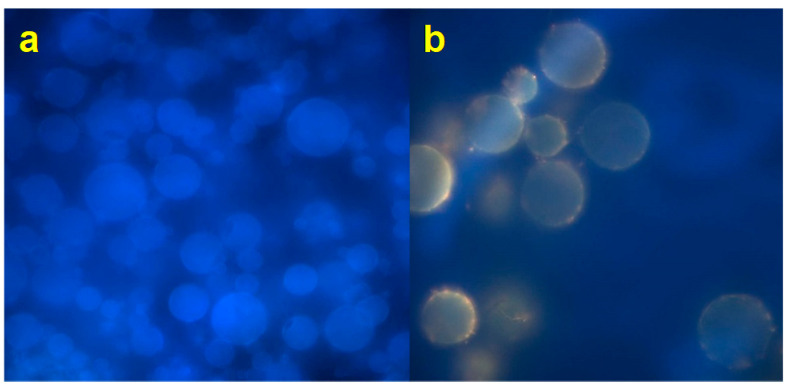
Fluorescence (FM) images of spray dried unloaded (EP_3_, (**a**) and loaded (AJEP_3_, (**b**)) microparticles.

As reported elsewhere, a well-encapsulated system in the amorphous state could more positively affect the retention of functional compounds than crystalline systems that could release compounds in a larger amount [48]. A fluorescence microscopy analysis was performed to better explain the distribution of the extract within the particles. Figure 5a shows blank microparticles (EP_3_), without extract loaded. The blue fluorescence is due to the DAPI used as a filter. Figure 5b shows the loaded microparticles AJEP_3_, with a clear yellow-orange fluorescence due to the presence of the extract. The extract is homogeneously distributed in the matrix and it has almost totally interacted with the matrix, resulting in being dispersed in an amorphous particulated structure. A small part of raw AJ is loaded on the surface or partially external to the particles. This behaviour is probably due to the small amount of the extract that does not interact with the matrix during the spray-drying process.

##### Thermal Analyses

**Figure 6:**
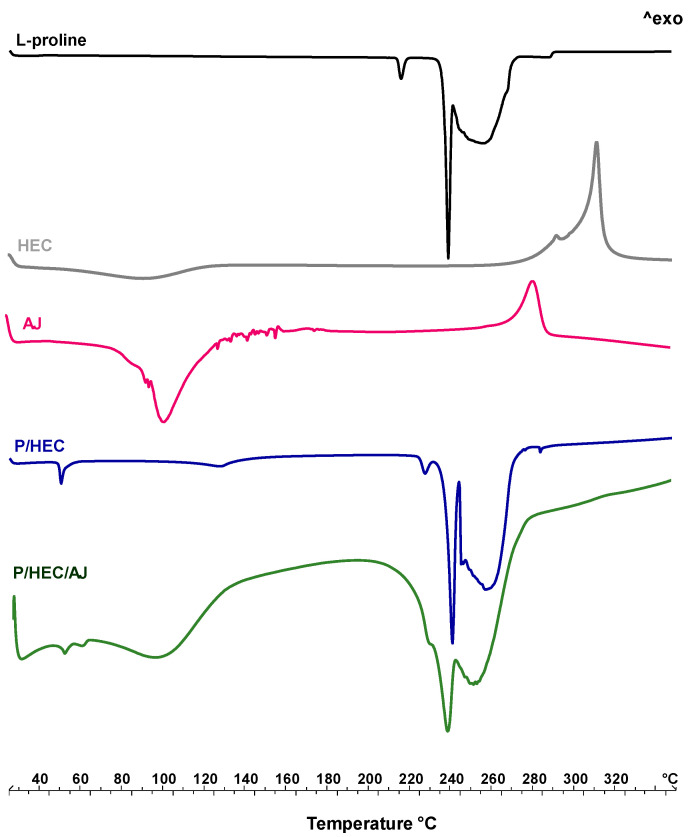
Differential scanning calorimetry (DSC) of raw materials (L-proline, black line, HEC, grey line, AJ, pink line), and physical mixture P/HEC (blue line) and P/HEC/AJ (green line).

Figure 6 shows the thermal behaviour of the raw materials, AJ extract, and their physical mixtures. L-proline (Figure 6, black line), used as the carrier of the system, has two main characteristic melting peaks at 217 °C and 246 °C, followed by the decomposition event [49]. The HEC thermogram (Figure 6, grey line) shows two main events, respectively, at above 100–120 °C on the baseline, related to the change of specific heat with a probable solid-solid transition associated with the dehydration and, at around 310 °C, the exothermic transition that generally anticipates the cellulose depolymerization [50].

AJ extract (Figure 6, pink line) exhibits the melting and degradation of different active components, mainly secondary metabolites, like phenols, present in the extract. These thermal events are distributed in a range between 85 °C to 190 °C with a final decomposition at above 325 °C [51,52].

The physical mixture P/HEC (Figure 6, blue line) shows the loss of residual moisture (peak at 60 °C) probably absorbed in the mixing phase and an anticipation of the proline melting peak at 235 °C due to the presence of HEC. No peaks related to chemical interactions have been detected.

In the thermal profile of P/HEC/AJ (Figure 6, green line), several endothermic events have been detected between 50 °C and 300 °C, highlighting the coexistence of peaks relating to the carrier employed and the raw extract [53,54].

**Figure 7:**
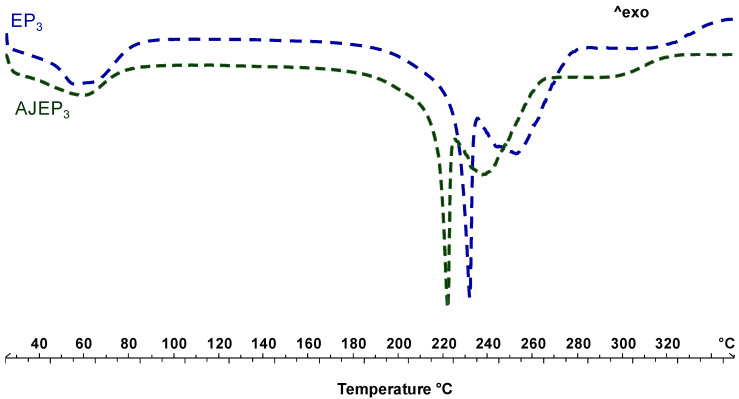
Differential scanning calorimetry (DSC) of blank (EP_3_, blue line) and loaded powder (AJEP_3,_ green line) at t_0_.

Figure 7 shows the thermal behaviour of unloaded (EP_3_, blue line) and loaded (AJEP_3_, green line) processed powders. The thermogram of EP_3_ shows the first endothermic event, visible at baseline, in the temperature range 50–100 °C due to the residual free water loss. The endothermic event starting at 220 °C is attributable to proline melting, which, according to previously reported data [22], reorganizes the residual crystalline material as the temperature increases and melts. The anticipation of the melting event (a shift of almost 20 °C) confirms the physical interaction between all of the matrix components during the spray-drying process.

AJEP_3_ thermal profile is almost like the EP_3_ profile, since the thermogram of loaded microparticles shows the absence of AJ extract endothermal events due to the wall protection, the physical interaction beetween matrix and extract, and homogeneous dispersion within the matrix.

##### Physical Stability

The stability study was carried out after six months (t_6months_) of storage in harsh conditions (40 °C, 75% RH ± 5%) in order to evaluate the maintenance of the physical characteristics of the produced powder over time giving greater usability of the product and also better understanding the added value of the observed technological improvements.

**Figure 8:**
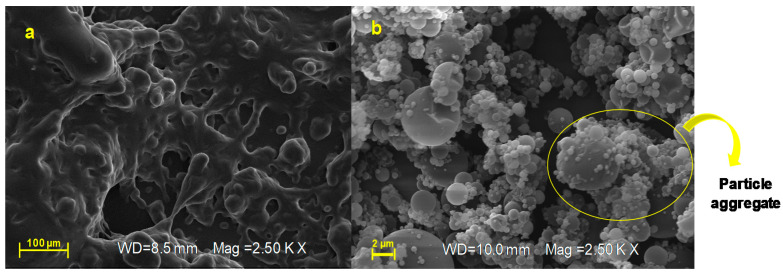
SEM micrographs (magnification: 2.5 KX, frame average, *n* = 1) of raw extract (AJ, (**a**)) and loaded powder (AJEP_3_, (**b**)) after six months (t_6months_) of harsh storage condition.

After six months of harsh storage conditions, the unprocessed extract AJ looks like a melted and sticky material that has completely lost the powder state (Figure 8a), while AJEP_3_ resulted in being unchanged (Figure 8b), remained morphologically stable, showing particles with a maintained spherical shape (Figure 8b). Otherwise, it is evident that small particles tend to form aggregates with larger ones.

**Figure 9:**
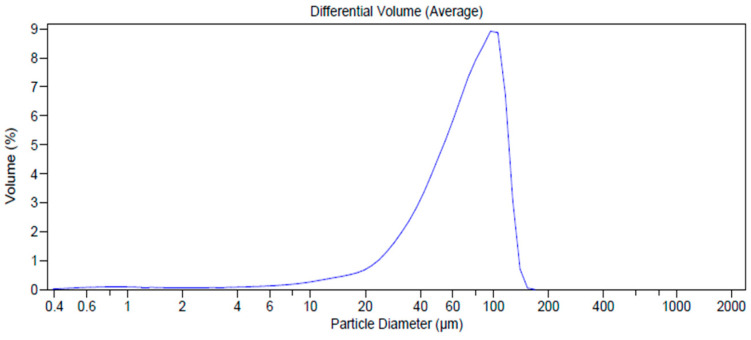
Dimensional distribution analysis (by Laser Light Scattering (LLS)) of AJEP_3_ after six months (t_6months_) of harsh storage conditions.

The phenomenon of aggregation tends to increase due to the influence of humidity, as shown by results from LLS analysis (t_6_ 71.51 µm, span 1.17, Figure 9 with respect to 18.46 µm t_0_, Figure 3d). However, the particles maintain both their morphology and solid-state without altering the overall appearance.

**Figure 10:**
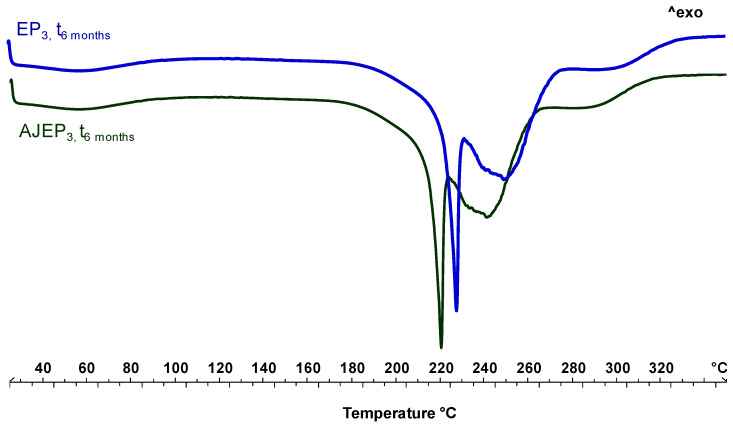
Differential scanning calorimetry (DSC) of AJEP_3_ (green line) and EP_3_ (blue line) after six months (t_6months_) of harsh storage conditions.

DSC thermal profiles of the powders after the storage period (Figure 10) confirmed the stability of the spray-dried products. The thermal trends of microparticulate powders are almost overlaid to the t_0_ profiles. The only slight detectable difference is the disappearance of the residual water at 60 °C and a slight increase in the intensity of the melting peak of the proline. This observation confirms that the mobility of the residual water during the storage led to a reorganization and generation of particle aggregates, as also showed by SEM and LLS analysis, which, in the thermogram, is manifested in a higher intensity melting peak under heating. Moreover, the absence of degradation peaks of active compounds in the extract supports the stabilization of AJ within the matrix [55].

#### 3.4.3. In Vitro Dissolution/Release Tests

**Figure 11:**
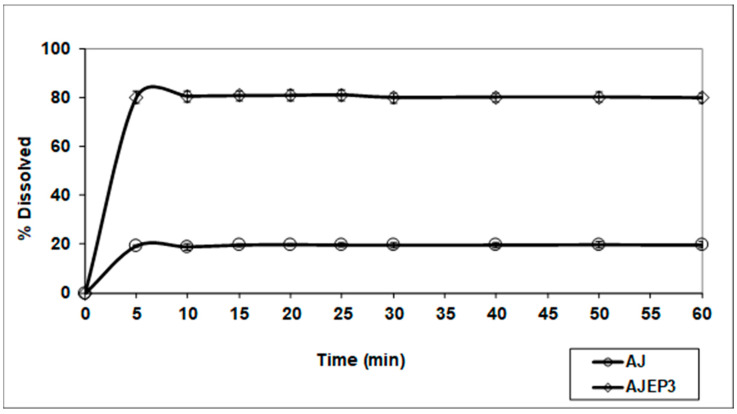
Dissolution/release profile of AJEP_3_ microparticles compared with AJ (unprocessed extract). The amount of the extract dissolved was measured calculating the content of teupolioside (**4**) by UV-method.

The USP II apparatus was selected in order to perform a preliminary study on the dissolution behaviour of spray-dried powder (AJEP_3_) as a conventional solid oral dosage form, with respect to the unprocessed AJ and the results in distilled water are reported in Figure 11.

**Figure 12:**
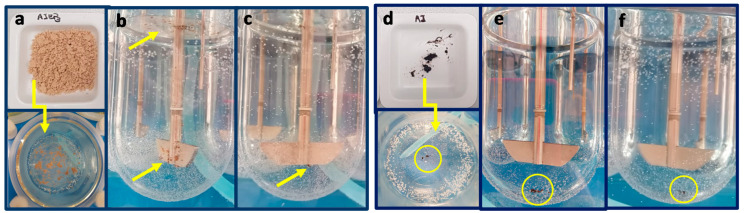
Pictures of water dissolution behaviour AJEP_3_ (**a**–**c**) and AJ raw extract (**d**–**f**): loading phase (**a**,**d**); one minute from the start of the test (**b**,**e**); and, five minutes from the start of the test (**c**,**f**).

Figure 12 shows that AJEP_3_ powder initially floats on the surface of the water and partially sticks to the blades of the instrument. However, after two minutes, it interacts with the dissolution medium, completely wets, and then dissolves. On the contrary, the unprocessed extract sinks into the vessel and remains almost completely undissolved (Figure 12).

An improvement of wettability due to the hydrophilic nature of HEC, combined with its swelling property, contributes to the observed enhancement of the dissolution rate. Moreover, the increase of the microparticle-water interaction due to both the smallest dimensions and amorphous physical state of microparticles enhances the total surface exposed to the solvent leading to an improvement of the water dissolution. Although 100% of the loaded dose of the extract is not dissolved, an evident improvement of the release and dissolution rate of the extract was obtained from AJEP_3_ profile (80% in five minutes) with respect to the unprocessed extract (20% in five minutes) (Figure 11). The small amount of AJEP_3_ that does not dissolve is probably due to the small part of the extract that does not fully interact with the matrix and remained outside the particles, as previously shown by FM images (Figure 5b).

### 3.5. Chemical Stability and Evaluation of the Functional Activity of AJEP_3_

The Actual Active Content (teupolioside %, *w*/*w*), the free radical scavenging activity against ABTS^•+^ and DPPH^•^ radicals, and the in-cell antioxidant properties of AJEP_3_ were analyzed in order to investigate whether the spray-drying process altered the chemical and functional stability of *Ajuga* components. All of the studies were conducted 72 h after the microparticles production and replicated after six months (for AAC and free radical scavenging tests) of storage at 40 °C (75% RH ± 5%). The obtained results (Table 2), shown as the designed matrix and the optimized microencapsulation process, did not affect the teupolioside content (from 2.70% at t_0_ to 2.50% at t_6months_), also preserving the extract efficacy against DPPH^•^ (from 130.11 µg/mL at t_0_ to 127.66 µg/mL at t_6months_) and ABTS^•+^ (from 0.22 mM Trolox/mg at t_0_ to 0.21 mM Trolox/mg at t_6months_). At the same conditions, the unprocessed extract (AJ) proved a significant (*p* < 0.05) reduction in teupolioside content (from 2.80% to 1.23%) and, consequently, a decrease of free radical scavenging activity, with EC_50_ from 135.78 ± 4.12 µg/mL to 161.71 ± 2.11 µg/mL and in TEAC value from 0.29 ± 0.01 mM Trolox/mg to 0.14 ± 0.06 mM Trolox/mg.

**Figure 13:**
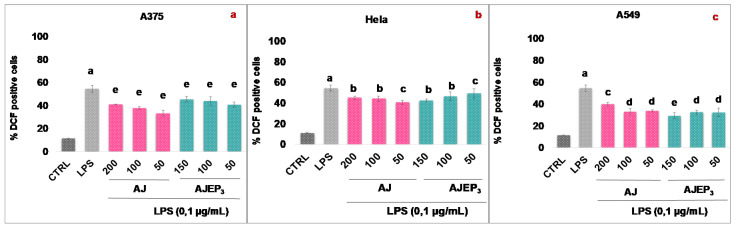
ROS formation was evaluated through the probe 2′,7′ dichlorofluorescein diacetate (H_2_DCF-DA) in A375 (**a**), HeLa (**b**), and A549 (**c**) cells. Unprocessed AJ (200–50 µg/mL) and µg of AJ in AJEP_3_ (150–50 µg/mL) were added for 1h and then for further 24 h exposed to lipopolysaccharides from *Escherichia coli* (LPS; 0.1 μg/mL). ROS production was expressed as mean ± standard deviation of the percentage of DCF positive cells of at least three independent experiments each performed in triplicate. The data were analyzed by one-way ANOVA, followed by Tukey’s test. different letters in the same column indicate a sgnificant difference, (**b**) *p* < 0.05, (**c**) *p* < 0.005, (**d**) *p* < 0.001, and (**e**) *p* < 0.0001.

Moreover, A375, HeLa, and A549 were treated with AJEP_3_ (at not cytotoxic concentrations) in order to verify whether the microencapsulation process modified the extract efficacy against ROS in human cancer cell lines. The obtained results (Figure 13) show that the spray-drying process did not affect the activity of AJ. In A375 and HeLa cells (Figure 13a,b) the effect of AJEP_3_ remained unchanged with respect to the unprocessed extract (AJ). In A549 cells (Figure 13c), 150 µg/mL of microencapsulated extract seems to be more active, as compared to the raw AJ in reducing the level of intracellular ROS.

These results imply that the higher percentage and rate of dissolution of AJEP_3_ in water than those of the raw extract AJ represent a great advantage both in aqueous cellular environment (e.g., AJEP_3_ was easily dissolved in the cell culture medium while AJ in DMSO), increasing the extract bioavailability, and to achieve a potential oral administration of the phytocomplex, as shown by the cellular assay and in the gastro-intestinal fluids where an enhanced dissolution may increase the bioavailability. When considered as a whole, the results suggest that AJEP_3_ may be proposed as an ingredient for a dietary supplement in the form of solid or liquid carrying 500 mg/mL of the powder, containing about 50 µg/mL (AEC = 9.69%, Table 1) of the bioactive AJ. However, further in vivo studies are necessary in order to confirm this assumption.

## 4. Conclusions

The methanolic extract (AJ) of *Ajuga reptans* L. and its major compounds characterized by chromatographic techniques, such as phenylpropanoids and iridoids, were able to reduce the Reactive Oxygen Species levels in cancer cell lines (melanoma, A375, cervical cancer, HeLa, and alveolar adenocarcinoma, A549), stimulated by *E. coli* lipopolysaccharide. Despite *Ajuga* extract being functional for reducing the damages of oxidative stress, which negatively affects cell viability, its practical application as dietary supplement in antioxidant-based therapies present challenges due to the poor organoleptic and physico-chemical characteristics of the extract. The choice of a multi-component polymeric matrix and spray-drying process parameters enhanced its biological and technological performance. The developed spray-drying method based on Proline/Hydroxyethylcellulose/Lecitin matrix led to obtaining a stable product in a microparticulate form with extended shelf-life and improved dissolution characteristics with respect to the raw material, suggesting higher feasibility in the oral administration. Moreover, the activity of the functional *Ajuga* extract in the HEC/P/L matrix was found to be retained, even after the spray-drying process. The made-up powder has the potential to be added into a liquid dosage form showing improved solubility during manufacturing process or into a solid dosage form showing enhanced AJ dissolution in aqueous biological fluids environment. The final product may be proposed as an active ingredient with good manufacturing characteristics and potentially enhanced bioavailability after oral administration in nutraceutics/dietary supplements useful in a disease prevention strategy.

## Figures and Tables

**Table 1:** Composition and characteristics of raw materials and spray-drying powders.

Sample	P/HEC g/100 mL	Water/EtOH Solvent System	L g/100 mL	AJ g/100 mL	Yield %	TEC ^a^ %	TAC ^b^ %	AEC ^c^ %	AAC ^d^ %	EE ^e^ %	d_50_ µm (span) ^f,e^
**AJ raw**	-		-	-	-	-	-	-	2.80 ± 0.5 ^d^	-	165.1 (2.02)
**HEC**	-		-	-	-	-	-	-	-	-	277.22 (1.60)
**P**	-		-	-	-	-	-	-	-	-	250.10 (1.71)
**EP_1_**	5.0/0.2	100/0	-		32.00 ± 1.14	-	-	-	-	-	-
**EP_2_**	5.0/0.2	100/0	0.2		51.28 ± 1.36	-	-	-			-
**EP_3_**	5.0/0.2	80/20	0.2	-	85.70 ± 1.91	-	-	-	-	-	102.93 (1.44)
**EP_4_**	5.0/1.25	H_2_O	-	-	10.31 ± 1.14						
**EP_5_**	5.0/0.5	H_2_O	-	-	20.66 ± 1.22						
**AJEP_1_**	5.0/0.2	100/0	-	0.55	32.76 ± 1.91	9.56 ± 0.31 ^f^	0.27 ± 0.05 ^f^	5.41 ± 0.63	0.14 ± 0.08	56.6	n.d.
**AJEP_2_**	5.0/0.2	100/0	0.2	0.50	41.00 ± 2.42	8.50 ± 0.64 ^f^	0.25 ± 0.04 ^f^	6.33 ± 0.71	0.18 ± 0.12	71.6	n.d.
**AJEP_3_**	5.0/0.2	80/20	0.2	0.58	71.50 ± 1.42	9.70 ± 0.37 ^f^	0.27 ± 0.02	9.69 ± 0.32	0.27 ± 0.06	99.9	18.46 (1.63)

P: proline; HEC: hydroxyethylcellulose medium viscosity; L: lecithin; AJ raw extract; ^a^ Theoretical extract content; ^b^ Theoretical Active Content; ^c^ Actual Extract Content; ^d^ Actual Active Content; ^e^ Encapsulation Efficiency; ^f^ Span value calculated as (d_90_ − d_10_)/d_50;_ All the data reported are the average of triplicate analyses ± SD.

**Table 2:** Actual Active Content (%) and free-radical scavenging activity of extract before (AJ unprocessed extract) and after microencapsulation process (AJEP_3_).

	t_0_	t_6months_	t_0_	t_6months_	t_0_	t_6months_
Samples	AAC% ^a^			DPPH Test EC_50_^b,c^		TEAC Test ^d,e^
AJ unprocessed extract	2.80 ± 0.40	1.23 ± 0.15 *	135.78 ± 4.12	161.71 ± 2.11 *	0.29 ± 0.01	0.14 ± 0.06 *
AJEP_3_	2.70 ± 0.06	2.50 ± 0.09	130.11 ± 0.04	127.66 ± 0.02	0.22 ± 0.03	0.21 ± 0.02
Teupolioside ^f^			9.18 ± 1.32	7.12 ± 0.80	0.64 ± 0.04	0.58 ± 0.04

*t*-test, means ± SD, * *p* < 0.05; ^a^ Actual Active Content (AAC): the content of teupolioside determined by HPLC-DAD; ^b^ EC_50_ ± standard deviation (data from three experiments in triplicate); ^c^ In a unit of µg of unprocessed AJ or µg of AJ in AJEP_3_; ^d^ TEAC value ± standard deviation (data from three experiments in triplicate); ^e^ TEAC value in unit of mM Trolox/mg of AJ or mg of AJ in AJEP_3_; ^f^ Positive control of the DPPH and TEAC test.
